# High sea surface temperatures driven by a strengthening current reduce foraging success by penguins

**DOI:** 10.1038/srep22236

**Published:** 2016-02-29

**Authors:** Gemma Carroll, Jason D. Everett, Robert Harcourt, David Slip, Ian Jonsen

**Affiliations:** 1 Department of Biological Sciences, Faculty of Science and Engineering, Macquarie University, Sydney NSW 2109, Australia; 2 Evolution & Ecology Research Centre, School of Biological Earth and Environmental Sciences, University of New South Wales, Sydney NSW 2052, Australia; 3 Taronga Conservation Society Australia, Bradleys Head Rd Mosman, Sydney NSW 2088, Australia

## Abstract

The world’s oceans are undergoing rapid, regionally specific warming. Strengthening western boundary currents play a role in this phenomenon, with sea surface temperatures (SST) in their paths rising faster than the global average. To understand how dynamic oceanography influences food availability in these ocean warming “hotspots”, we use a novel prey capture signature derived from accelerometry to understand how the warm East Australian Current shapes foraging success by a meso-predator, the little penguin. This seabird feeds on low trophic level species that are sensitive to environmental change. We found that in 2012, prey capture success by penguins was high when SST was low relative to the long-term mean. In 2013 prey capture success was low, coincident with an unusually strong penetration of warm water. Overall there was an optimal temperature range for prey capture around 19–21 °C, with lower success at both lower and higher temperatures, mirroring published relationships between commercial sardine catch and SST. Spatially, higher SSTs corresponded to a lower probability of penguins using an area, and lower prey capture success. These links between high SST and reduced prey capture success by penguins suggest negative implications for future resource availability in a system dominated by a strengthening western boundary current.

To understand how changes in the physical properties of oceans affect food webs, it is essential to develop appropriate indicators of resource availability. Remote sensing of environmental data including sea surface temperature (SST) and chlorophyll *a* provides valuable insight into the processes driving spatial and temporal changes in primary productivity^1^. Linking these changes to outcomes for mid-and upper-trophic level predators is more challenging, despite the importance of this information for predicting ecosystem responses to climate change^2^. As it is difficult to directly measure prey abundance on scales relevant to marine predators, studies have often used tracking technology to identify correlations between oceanography, primary productivity and predator foraging behaviour^3^,^4^. However, without a measure of foraging success, it is difficult to estimate how much prey animals encounter and consume while foraging under different environmental conditions.

Accelerometry has shown great potential to provide fine-scale information on the activity of wild animals. Machine learning algorithms have been applied to recognise patterns in acceleration data, providing robust links between the behaviour of animals and their movement profiles, such that behaviour can be determined remotely^5^. One application of these behaviour classification methods has been to identify movement patterns associated with feeding^6^,^7^,^8^. The next step in this rapidly evolving field is to use information on prey capture events to answer important ecological questions relating to resource availability that were previously difficult to address. By linking foraging success to physical oceanography, we can gain insight into the mechanisms dictating resource availability and predict if and how marine predators and their prey are vulnerable to environmental change.

Western boundary currents are a significant source of global heat transport, advecting warm water from the tropics to temperate latitudes^9^. Although these systems are considered nutrient poor compared with cold eastern boundary currents, they generate eddies^10^ that drive nutrient upwelling^11^. Subsequently, they are able to support fisheries^12^ and populations of marine predators such as seabirds (e.g.)^13^. Western boundary currents are strengthening, increasing the pole-ward penetration of warm water. For example, the East Australian Current (EAC) is the western boundary current of the South Pacific Gyre, and its intensification is causing regional sea surface temperature (SST) to rise much faster than the global average^14^. The global significance of changes to currents means that it is important to understand how their physical processes drive variation in productivity^15^, and to identify the effects of this variability at all trophic levels.

Seabirds provide a useful model to examine the effects of environmental change on resources, as they are highly responsive to fluctuations in prey availability^16^. The little penguin (*Eudyptula minor*) is the world’s smallest species of penguin and has a breeding colony at Montague Island, off southeast Australia (774 ± 61 breeding pairs on the southern part of the island in 2015; Peter Fullagar, unpublished data). Montague Island is situated halfway across the continental shelf, with surrounding waters heavily influenced by the dynamics of the EAC and its eddy field (Fig. 1A). To assess the consequences of variation in EAC penetration on the amount of prey (low trophic level species, or “forage fish”, including small pelagic fish, krill and squid) caught by penguins, we used a prey capture signature derived from their acceleration profiles^7^. As little penguins are highly constrained in the range and duration of foraging trips during the breeding season, we determined mechanistic links between foraging success and the environment on fine spatial (<25 km) and temporal (<10 day) scales.

Specifically, our aims were to: 1) understand the temporal variability of prey capture success by little penguins determined using accelerometry, and assess how this variability was related to the EAC; 2) understand spatial variability in foraging location determined using GPS, and assess how environmental features dictate the way that penguins use available foraging habitat; 3) assess spatial variability in prey capture success determined from GPS, accelerometry and depth recorders in relation to the EAC. We discuss the insight that our findings give into the relative availability of low trophic level species to predators in the context of western boundary current intensification.

## Methods

### Fieldwork

The field study was conducted on Montague Island (−36.253°, 150.227°), 9 km off the southeast coast of New South Wales, Australia, in September, November and December 2012–2014. This period covers the peak of the little penguin’s breeding season, which can be highly asynchronous^17^. Adults show bi-parental care during incubation and rearing offspring. During incubation, foraging trips are on average 3.5 days in length^18^. When offspring are young (<2 weeks) one parent stays behind to guard the chicks while the other goes to sea usually for a single day, and as the chicks grow both parents go to sea simultaneously, often for multiple days. When conditions are good, little penguins can raise two clutches in succession^19^. Sampling periods comprised 6–15 days tracking penguins in each month.

The night before a penguin went to sea, it was caught in its nest box and equipped with a GPS logger (CatTrack, South Carolina, USA) modified with epoxy resin to withstand pressure at depth. The loggers were inserted into heat shrink tubing, then attached to feathers on the lower back with cloth tape (Tesa, Hamburg, Germany), positioned so as to reduce drag but not impede tail movement. These tags were 43 mm in length, 27 mm in width and 13 mm in height, and weighed 55 g in air and 17.4 g in seawater. A range of sampling frequencies was used over the study period to adjust the trade-off between battery life and spatial resolution. For penguins expected to perform single day trips (birds rearing small chicks), sampling frequency ranged from 7 – 45 s. For birds expected to perform multiple day trips (incubating birds and birds with larger chicks), the sampling frequency ranged from 30–115 s, however at these sampling frequencies the loggers were still rarely able to record complete multiple days, so spatial analyses were restricted to single day trips. When penguins returned from a foraging trip they were recaptured in their nest boxes, loggers removed and the penguin was weighed in a calico bag using a spring balance scale (Pesola, AG Switzerland).

Accelerometer data loggers (G6a and G6a + , CEFAS Technology Pty Ltd, Suffolk, UK) were attached immediately in front of the GPS units (towards the head) on the middle back for a subset of penguins from November 2012. These tags were 40 mm in length, 28 mm in width and 15 mm in height, and weighed 7.8 g in air and 2.3 g in seawater. The accelerometers recorded acceleration in 3 axes: anterior-posterior (surging), lateral (swaying) and dorso-ventral (heaving) with a range of + /−2 g. The accelerometers recorded depth, temperature and acceleration and were programmed in two modes: “shallow” mode (<1.5 m: 1.5% of the full scale pressure range) where parameters were recorded every 10 seconds, and “dive” mode (>1.5 m) where the same parameters were recorded at 30 Hz.

Combined tag weight for penguins that were equipped with both accelerometers and GPS was 62 g in air and 19.7 g in seawater, which is < 5% and < 2% of mean bodyweight (~1100 g) respectively. Handling time was kept to a minimum, and in most cases was less than 5 min for both deployment and retrieval of devices. All animal research protocols were carried out in accordance with guidelines approved by the Macquarie University Animal Ethics Committee (Animal Research Authority 2011/14).

### Prey capture signature

We previously developed a motion signature to identify prey capture by wild penguins at sea, using a support vector machine algorithm that identified prey handling by wild penguins with a false positive rate of 0.09%. A thorough description of this method and its validation in the wild is detailed in Carroll *et al*.^7^. We showed that dives during which prey capture occurred were longer in duration, deeper, had longer bottom times, more undulations in the bottom phase of the dive and faster ascent and descent rates, consistent with predictions from foraging theory and previous empirical studies of marine predator foraging ecology^20^,^21^,^22^.

### Analysis

#### Temporal variability in prey capture success

We assessed temporal patterns of prey capture success by breeding little penguins between November 2012 and December 2014. All available accelerometry profiles were used in this analysis, from both single and multiple day foraging trips. The penguins in this study were not individually marked, so it is not known whether they were resampled in multiple years. However, only 9 birds of the same sex from the same nest were sampled more than once (13%). We used the mean number of prey caught per 90 min period across a foraging trip as an index of prey capture success for that trip. As we might expect penguins to adjust their foraging effort to the availability of prey, we also calculated a measure of catch per unit effort (CPUE), which was the mean value across a foraging trip of the number of prey caught per 90 min window as a function of time spent diving below 1.5 m within that window. We used the mean value over discrete time windows rather than a daily value, as this allowed for comparison among foraging trips of different lengths. We tested a range of time windows (1, 5, 10, 20, 30, 45, 60 90, 120 and 240 min) on a subset of eight pooled single and multiple day foraging trips and found that 90 minutes was the window that minimised the standard deviation of the mean foraging trip CPUE (see Figure S1 in Supplementary Material). For trips longer than one day, we removed all 90 min intervals that occurred at night, as penguins do not forage after dark^23^. In this study time spent diving was usually < 1 min per 90 min window after sunset.

To assess the temporal influence of the EAC on foraging success, we first described relationships between different environmental variables to find a suitable means of representing EAC penetration. We obtained measurements of satellite-derived SST (MODIS-Aqua), chlorophyll *a* (OC3) and geostrophic velocity (derived from altimetry from NASA/CNES (Jason-1 and 2) and ESA (ENVISAT) satellites) from the Integrated Marine Observing System (IMOS) Data Portal (http://imos.aodn.org.au/imos/). Daily SST and Chlorophyll *a* data were obtained at a location 5.5 km offshore (east) from Montague Island (36.26°S, 150.29°E) in November and December 2012–14 and September 2013–14 (accelerometry data was not collected during September 2012). This offshore location is halfway between Montague Island and the edge of the continental slope where the EAC is centred^24^, and therefore should provide an index of the incursion of offshore EAC waters onto the shelf. We used oceanographic data from this location after comparison with data taken from a location 3 km inshore (west), halfway between Montague Island and the mainland (36.26°S, 150.19°E), and values averaged over the penguin foraging area. The single offshore location better captured the variability associated with EAC penetration, as indicated by the fact that Akaike’s Information Criterion (AIC) scores were lowest with offshore SST, when the same models were run with each of these variables in turn (see below for model details). North-south geostrophic velocity was obtained at the closest pixel to the SST data (36.2^o^ S, 150.4 ^o^E) and rotated 19-degrees to be in the alongshore direction.

Using linear regression we examined the relationship between offshore SST, chlorophyll *a* from the same location, and geostrophic current velocity (see Figure S2 in Supplementary Material). As expected, a stronger alongshore current was correlated with warmer water (adjusted R^2^ = 0.17, p < 0.0001) and chlorophyll *a* concentration decreased with warmer water (adjusted R^2^ = 0.36, p < 0.0001). Due to these correlations we chose not to model the effect of more than one of these environmental variables on foraging success simultaneously, although they are each likely to explain a portion of its variance. We chose to use offshore SST as a single proxy for penetration of the EAC, as the EAC brings warm water down Australia’s southeast coast from the tropics in a series of highly variable mesoscale eddies, rather than a continuous southward-flowing stream^10^,^24^. Increasing temperature brought by these warm influxes is likely to be of greater biological relevance to larger organisms than for example, an increase in the speed of the current. Furthermore, the measurement of SST in coastal systems is more reliable than measurement of chlorophyll *a*, and occurs at a higher sampling frequency (daily) compared to geostrophic velocities, which are calculated from satellite altimetry data and collated and interpolated over a 10-day cycle.

To characterize seasonal patterns of EAC penetration, we plotted a time series of daily SST values offshore from Montague Island for 2012–2014, and applied a 10-day rolling mean to smooth the data. To place our findings in a longer-term context, we calculated the mean SST for each calendar day over a period of 11 years (2003–2014). We also calculated the mean SST for each month that penguins were tracked.

We used generalised additive models (GAMs) with Gaussian error distributions to estimate the relationship between SST and our two measures of foraging success (mean number of prey caught per 90 min window and CPUE). For each penguin foraging trip, we averaged SST over a four-day window centred on the trip dates. If the foraging trip was longer than four days, we used the mean over the length of the trip. This helped to reduce gaps in satellite data arising from cloud cover, but was still relevant for penguins foraging on short temporal scales.

#### Spatial variability in foraging location

We used locations recorded by GPS loggers to determine the relationship between spatial habitat use and SST. For these analyses we only used single day foraging trips, as these were comparable in terms of the maximum distance that the penguins travelled from the island (~25 km per day). By focusing on single day trips, we were also able to use the most complete GPS tracks, as tracks of multiple day foraging trips were often incomplete due to limitations in GPS battery life on these small loggers.

To determine the relationship between foraging location and SST, we created a grid with 1 km^2^ cells spanning the penguins’ potential single day foraging range (25 km north and south of Montague Island and east to the shelf edge). The 1 km^2^ grid was the finest resolution available for the remote-sensed MODIS-Aqua SST data. We averaged SST over the days when we tracked penguins (e.g. if there were penguins tracked on the 3^rd^, 4^th^, 6^th^, 8^th^ and 11^th^ of September, we averaged gridded SSTs for these days). We chose this method after testing two others that masked relationships between SST and foraging success: a) taking the mean of SST for all days within the range of dates that penguins were tracked (e.g. 3^rd^ – 11^th^ September) and b) averaging over 15 days regardless of the tracking date range (15 days being the longest tracking period).

We spatially binned locations recorded by the GPS tags onto the same 1 km^2^ grid, resulting in counts of locations within each grid cell that we summed for each sampling period (Sep, Nov & Dec 2012–14). To avoid artificially over-sampling in some areas where penguins rested at the surface, and under-sampling in other areas where gaps were recorded in the GPS data due to the penguins spending more time underwater, we linearly interpolated the raw GPS location data at regular 10 min intervals. Interpolation also allowed us to homogenise the different GPS sampling frequencies used during the study period. We determined a 10 min sampling frequency to be the most appropriate as little penguins travel at a mean speed of 1.8 m/s^25^, and therefore move, on average, ~1 km every 10 minutes. Thus, 1 km^2^ areas that penguins moved through without foraging would get on average a single count (or fewer if the penguin was moving more quickly), whereas areas where they encountered prey and engaged in area restricted search were likely to contain substantially more observations.

We analysed the relationship between SST and penguin location counts using a hurdle model^26^. This model accounts for the zero-inflation present in the count data (there were large areas where the penguins did not go and hence many zero counts in grid cells) as well as over-dispersion (some cells had few detections while others had many). The hurdle model uses two processes to model data. The first assesses the relationship between the predictor variable (in this case SST) and counts (the number of times penguins were observed in a cell). The second assesses the relationship between the predictor variable (SST) and the zero observations (presence/absence of penguins in a cell) using a binomial distribution. The model assumes that SST might differentially affect a) whether penguins do or do not go to an area at all (habitat suitability) and b) how much time they spend there (habitat quality).

To test whether penguins are responding to relative SST or absolute temperatures, we assessed both SST and SST anomaly (deviance of SST for each 1 km^2^ grid cell from the mean SST of all grid cells). We compared three measures (SST, SST anomaly (continuous predictor) and SST anomaly (binary predictor; warmer or cooler than the mean)) in separate hurdle models and compared them using Akaike’s Information Criterion (AIC), to best capture the relationship between SST and both penguin presence/absence and the number of observations within grid cells. We created a visual representation of the relationship between foraging location and SST by overlaying raw GPS tracks on a map with spatial SST anomaly (pixels were coloured according to how much they deviated from the mean temperature of the study area).

#### Spatial variability in prey capture success

We assessed the spatial distribution of prey capture events in relation to SST. We performed a linear interpolation between GPS locations at 1 s intervals to integrate the accelerometry record and the GPS tracks. When a location was determined for each prey capture event, we used only these locations in the analysis. We used the same 1 km^2^ grid and counted prey capture events recorded in each cell. We analysed only the cells in which prey capture occurred, using a GAM to assess relationships between prey capture density and SST. We then examined the time series of SST encountered by penguins through the course of single day trips (mean SST of grid cells visited per 10% increment by all penguins within a sampling period) in relation to the prey caught through the course of single day trips. We also determined the mean depth of prey capture events during each foraging trip, and the mean distance of prey captures from the colony during each foraging trip, and assessed how these varied intra- and inter-annually in relation to offshore SST (described previously).

All analyses conducted in this study were performed in the R statistical programming framework^27^.

### Ethics statement

The Macquarie University Animal Ethics Committee approved all animal research protocols, which were undertaken in accordance with guidelines set out by Australian law (Animal Research Authority 2011/14).

## Results

### Oceanography

**Figure 1:**
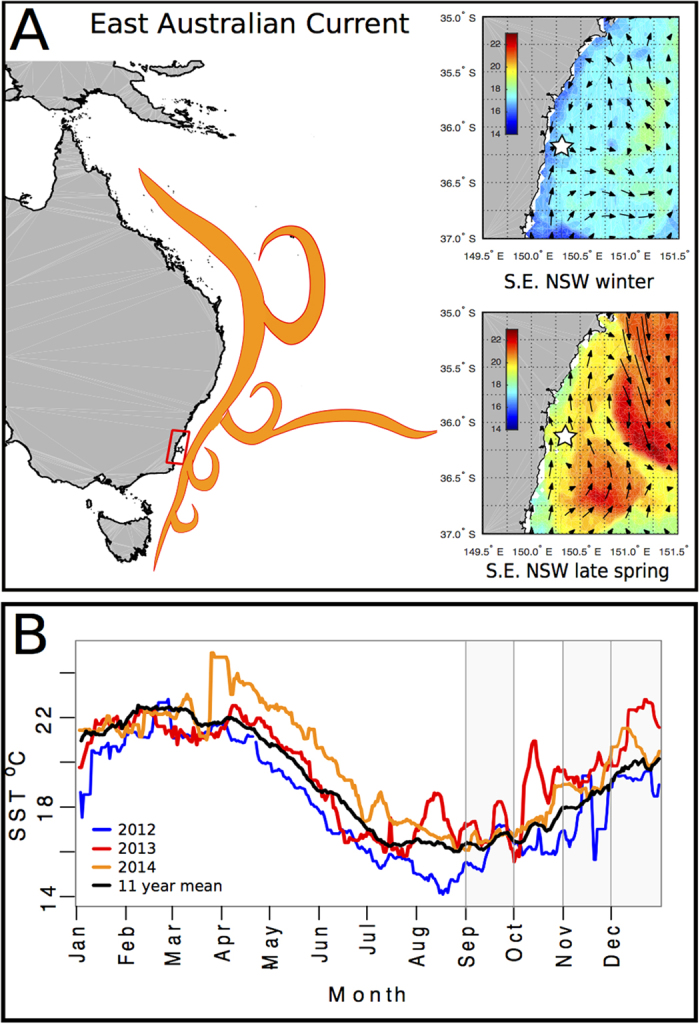
(**A**) Schematic showing the general characteristics of the East Australian Current (left). The area of this study (southeast NSW) is indicated by the red box, and the white star marks the location of Montague Island. On the right are examples of typical winter (top) and late spring (bottom) sea surface temperature and current directions in southeast NSW around Montague Island (white star). Inset maps were produced in MATLAB R2014b using data available from http://imos.aodn.org.au/imos/. (**B**) Annual time series of sea surface temperature measured offshore from Montague Island during 2012, 2013 and 2014 with a rolling 10-day mean. Grey windows represent the months (Sep, Nov, Dec) in which penguins were tracked from 2012–2014.

Penetration of the East Australian Current was variable over the three study years (Fig. 1). In 2012, SSTs were almost always lower than the long-term average. By contrast, both 2013 and 2014 had summer SSTs that were generally warmer than average. In particular, SSTs appeared anomalously high in 2013 during the penguin breeding season, with high variability and strong pulses of warm water penetrating the study region. The timing of the onset of warm water penetration in 2013 was notable, with an unusual infiltration of warm water in August resulting in SSTs more than 1 °C warmer than the long-term mean. In October 2013 there was a spike in SST of almost +4 °C from the mean and in December 2013 an increase of around +3 °C. 2014 was less variable, but there was an anomalous spike of +1 °C in late October to mid-November 2014, and another of +1.5 °C in December 2014.

### Temporal variability in prey capture success

**Figure 2:**
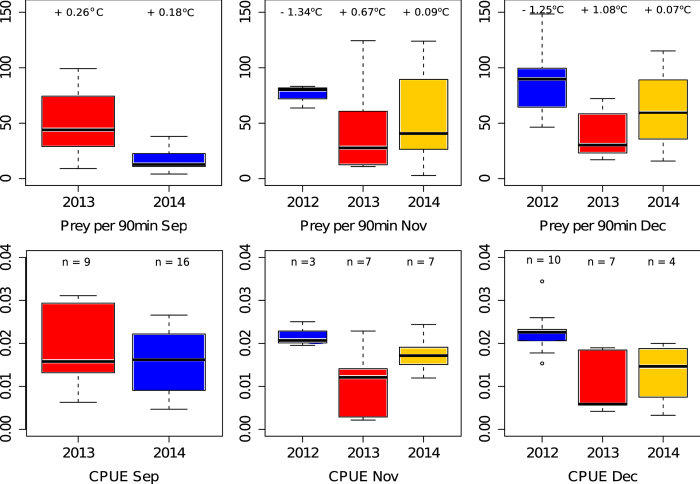
Mean prey caught per 90 min window and mean CPUE (prey caught per minute spent diving below 1.5 m) per 90 min window by month in 2012, 2013 and 2014. Box plots are coloured according to the SST relative to the mean SST of that month in the other two study years (red being the warmest of the 3 years for each month, blue being the coldest). Sample size and deviation from the mean monthly temperature (^o^C) are noted on the prey capture plot (top row) and samples sizes are shown on the CPUE plot (bottom row).

We obtained accelerometry profiles for 63 penguin foraging trips between Nov 2012 and Dec 2014 (Nov 2012 n = 3; Dec 2012 n = 10; Sep 2013 n = 9; Nov 2013 n = 7; Dec 2013 n = 7; Sep 2–14 n = 16; Nov 2014 n = 7; Dec 2014 n = 4). The mean number of prey caught per 90 minute period varied both intra- and inter-annually (see Fig. 2) (mean ± s.e.: Sep 2013 = 51.65 ± 10.97, Sep 2014 = 19.18 ± 3.97; Nov 2012 = 75.74 ± 6.10, Nov 2013 = 44.22 ± 15.74, Nov 2014 = 57.04 ± 17.47; Dec 2012 = 88.09 ± 9.22, Dec 2013 = 40.86 ± 8.52, Dec 2014 = 63.24 ± 20.77). We tested whether inter-annual differences were significant using a generalised linear model (GLM) for each of the three months, assessing the relationship between 90 min prey capture success and year. Where there were 3 years (November and December), 2012 was the reference year as it always had the highest prey capture success. In September, 2014 was significantly worse for prey capture success than 2013 (−32.47 ± 9.69, t = −3.350, p = 0.003). In November, there were no significant differences, and in December, 2013 had significantly lower prey capture success than 2012 (−47.23 ± 14.64, t = −3.22, p = 0.005). CPUE followed the same general trend as the 90 min catch data, although in September penguins spent less time actively foraging relative to the number of prey caught, thereby reducing the difference in CPUE between 2013 and 2014.

In November and December, the monthly SST anomaly (SST relative to mean SST for that month across the 3 study years) showed a broad correlation with prey capture success as determined by both 90-minute prey capture and CPUE metrics. 2012 was the year with lowest SSTs (Nov = −1.34 °C; Dec = −1.25 °C relative to the mean), and saw the highest foraging success. 2013 was anomalously warm, (Nov = +0.67 °C; Dec = +1.08 °C) and saw the lowest foraging success. 2014 was intermediate in both SST and foraging success (Nov = +0.09 °C and Dec = +0.07 °C). In September this trend was reversed with 2013 being warmer with higher prey capture success, although there was low variability in SST between the two years (Sep 2013 = +0.26 °C, Sep 2014 = +0.18 °C).

**Figure 3:**
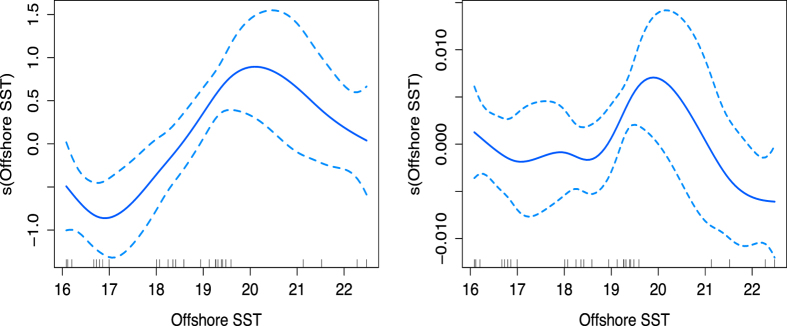
Generalised additive model relationships between log transformed prey capture by penguins per 90 min and offshore SST (left); and catch per unit effort (number of prey caught by penguins/amount of time spent diving > 1.5m) & offshore SST (right).

Offshore SST had a quadratic relationship with both log transformed prey captures per 90 min and CPUE (Fig. 3). For the 90 min prey capture data, low SSTs (16 °C −18.5 °C) corresponded to the lowest prey capture success. A peak in prey captures occurred at around 20 °C before dropping off (GAM R^2^ = 0.31, F = 5.79, p = 0.0002). There was no strong relationship between CPUE and SST between 16 °C and 19 °C, when again the model showed a strong peak in CPUE around 20 °C before a drop off between 21 °C and 22 °C (GAM R^2^ = 0.17, F = 2.57, p = 0.03).

### Spatial variability in foraging location

For the habitat use analysis, we analysed GPS tracks from a total of 112 single day penguin foraging trips in September, November and December 2012–14 (Sep 2012 n = 31; Nov 2012 n = 6; Dec 2012 n = 10; Sep 2013 n = 15; Nov 2013 n = 19; Dec 2013 n = 12; Sep 2014 n = 9; Nov 2014 n = 6; Dec 2014 n = 4). 82% of 1 km^2^ grid cells that penguins could have visited during single day trips did not contain observations of penguins, indicating that penguins were selective in their habitat choice, foraging in similar locations within a given time period.

**Figure 4:**
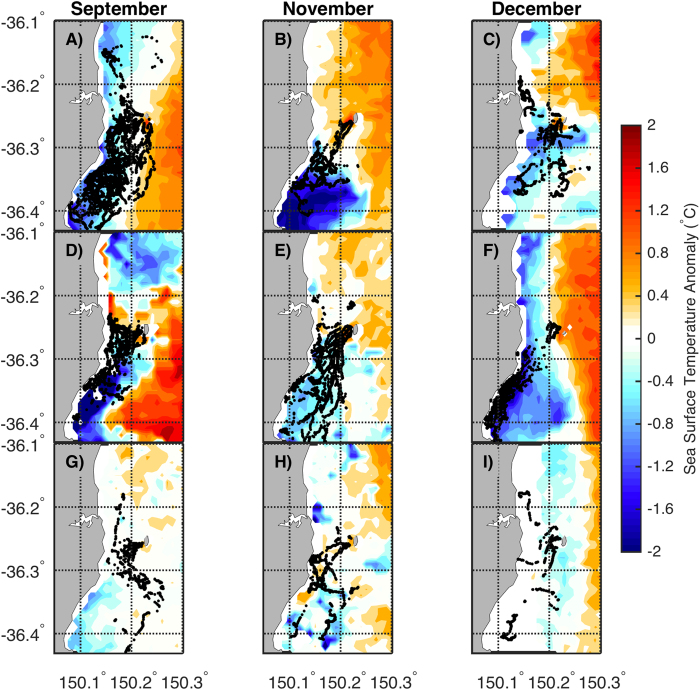
Raw GPS tracks of penguins performing single day foraging trips in relation to sea surface temperature anomalies (SST of 1 km^2^ grid cell – mean SST of all 1 km^2^ grid cells in study area). Top row is 2012, second row is 2013 and bottom row is 2014. Plot regions represent the area gridded on a 1 km^2^ scale for spatial analyses. Maps were produced in MATLAB R2014b using data available from http://imos.aodn.org.au/imos/.

Penguins appeared to seek out water that was colder than the mean of all available habitat (Fig. 4). When a grid cell was colder than average, we were 42% more likely to observe a penguin than if the grid cell was warmer than average (26% of cells colder than average recorded penguin presence c.f. 15% of cells that were warmer than average). Similarly, the time that penguins spent in a cell was related to its SST anomaly: areas colder than average had 50% higher counts than areas warmer than average (cold = 2.76, warm = 1.38).

The hurdle model using SST averaged over the sampling days as a predictor performed better than models using SST anomaly either as a continuous or binary variable (see Table S1). The count part of the hurdle model, which explains variation in the number of times penguins are observed in a cell, showed that lower counts were recorded as SST increased (SST estimate = −0.02, S.E. = 0.01, Z value = −3.00, P value = 0.003). For the zero part of the hurdle model, which explains variation in whether penguins were observed in a cell at all, showed that as SST increased, penguins were increasingly less likely to visit that cell (SST estimate = −0.28, S.E = 0.03, Z value = −10.45, P value = < 0.0001).

### Spatial variability in prey capture success

**Figure 5:**
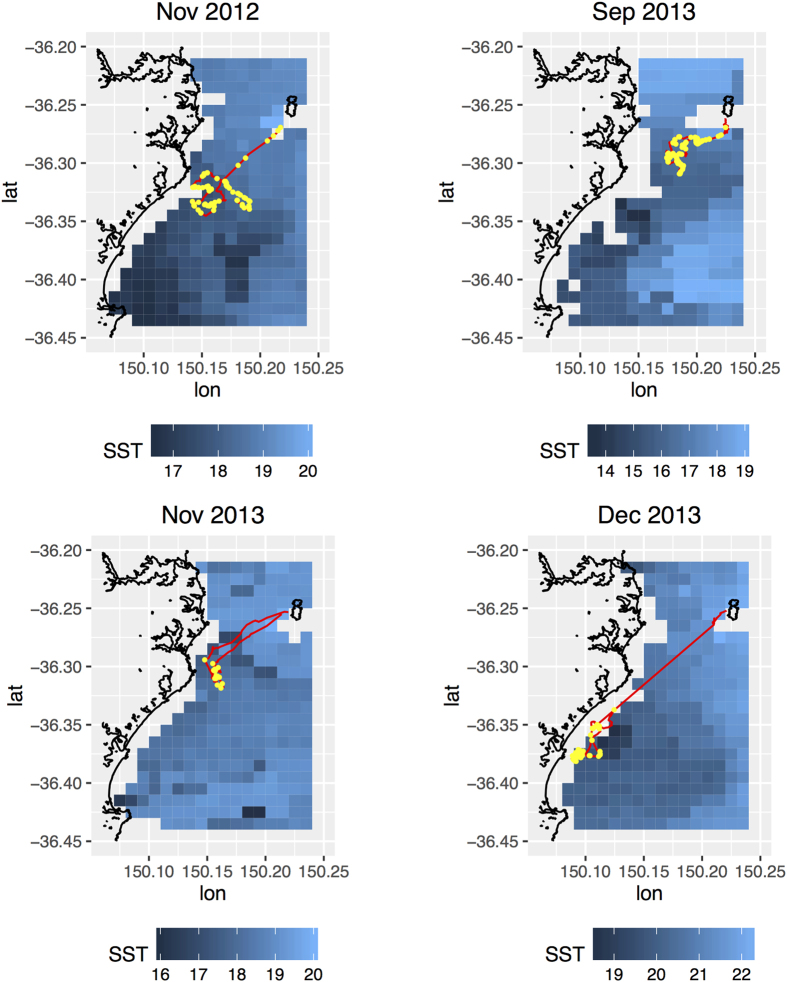
Sample foraging trips by little penguins in relation to gridded sea surface temperature (http://imos.aodn.org.au/imos/), showing foraging trajectory (red) and prey capture locations (yellow). Panels have different scales in order to highlight penguin habitat selection relative to the distribution of sea surface temperatures within each period. Plot created using *ggplot2*^64^ in R version 3.2.3^27^.

To assess the effect of SST on the spatial distribution of prey captures, we integrated accelerometry profiles and GPS tracks of 50 complete single day penguin foraging trips in November and December 2012–14, and September 2013–14 (Nov 2012 n = 3; Dec 2012 n = 10; Sep 2013 n = 6; Nov 2013 n = 8; Dec 2013 n = 8; Sep 2014 n = 7; Nov 2014 n = 4; Dec 2014 n = 4). Sample penguin tracks with prey capture locations in relation to gridded SST are shown in Fig. 5. These illustrate habitat selection by the penguins, with penguins tending to forage in cooler waters, particularly in warmer months (e.g. in December 2013). Examples of gridded prey capture densities by all penguins within each month in 2013 are shown in Figure S3. There were signs of a relationship between SST and the number of prey caught within a 1 km^2^ grid cell at the coldest and warmest temperatures observed during this study (Figure S4). The GAM showed that the highest density of prey captures occurred when penguins were in areas with the lowest recorded temperatures (~13.5 °C). Prey capture success was variable at intermediate SST and fell when temperatures were > 20 °C. Even after removing the effect of unsuitable habitat where there may be no prey at all, there was an effect of SST on the spatial distribution of prey captures. However, the modelled relationships retained some uncertainty and the amount of variance explained was low (GAM R^2^ = 0.11, F = 4.05, p < 0.0001).

**Figure 6:**
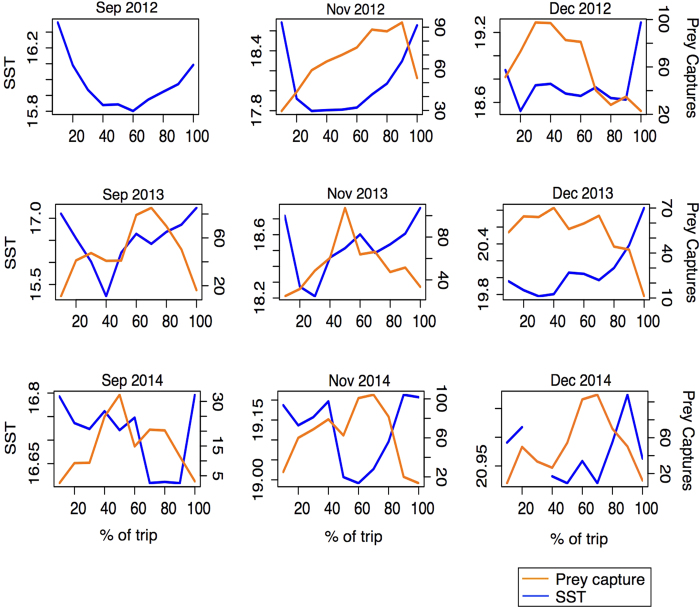
SST (blue lines) and prey capture events (orange lines) as a function of time elapsed in a foraging trip (each trip was divided into 10% quantiles). SST values are the mean of the SSTs encountered by all GPS-equipped penguins in 1 km^2^ grid cells in each 10% interval. Prey capture values are the mean of the number of prey captures recorded by accelerometer- and GPS-equipped penguins in each 10% interval.

The GAM only assessed the effect of SST on prey capture success in areas where prey capture occurred. To assess the relationship between prey capture and the SSTs encountered over the course of a foraging trip, we plotted a time-series of the mean SST of grid cells visited by penguins in 10% increments of trips within a sampling period (September, November and December 2012–14). We then overlaid the mean number of prey captures identified using accelerometry within the same 10% increments within a sampling period (November and December 2012–14, September 2013–14) (see Fig. 6).

This analysis confirmed that penguins seek out areas with lower relative SSTs, with encountered temperatures always decreasing during a foraging trip before increasing again towards the end of a journey. It also showed that in December 2012–14 and November 2012 and 2014, there was good agreement between the spatial distribution of prey capture success and SST, with areas/periods of lowest SST encountered by penguins on a foraging trip related to the highest prey capture success. However, in September 2013–14 and November 2013 there was no clear relationship between spatial prey capture and SST distribution.

In order to explore whether subsurface water properties such as thermoclines might affect little penguin foraging success differently from SST, we assessed the relationship between the mean SST in a 1 km^2^ grid cell and the mean temperature at the point of prey capture in the same grid cell, calculated from the temperature sensors on board the accelerometers. We found a near linear, 1:1 relationship between SST and prey capture temperature above 16 °C (GAM R^2^ = 0.53, F = 28.02, P < 0.0001) (Figure S5). This is unsurprising, as prey capture by penguins generally occurred in the upper part of the water column, with the mean depth of prey captures across foraging trips being less than 10 m in all months (mean (m) ± s.e.: Sep 2013 = 4.26 ± 0.20, Sep 2014 = 4.57 ± 0.35; Nov 2012 = 6.77 ± 0.81; Nov 2013 = 8.46 ± 0.59, Nov 2014 = 8.49 ± 0.70; Dec 2012 = 9.93 ± 0.82, Dec 2013 = 9.18 ± 0.99, Dec 2014 = 6.89 ± 1.49). There were no obvious patterns between depth of prey capture and the relative SST for each month (Figure S6).

The mean distance from the colony at which penguins caught prey ranged from 8 km to 16 km (Sep 2013 = 8.18 ± 0.79, Sep 2014 = 7.28 ± 0.86; Nov 2012 = 8.55 ± 0.91; Nov 2013 = 13.32 ± 2.30, Nov 2014 = 8.18 ± 0.49; Dec 2012 = 8.07 ± 1.04, Dec 2013 = 15.68 ± 0.84, Dec 2014 = 12.94 ± 3.47). It appears that prey captures occurred further from the colony when SST was relatively warm (Figure S6). This was confirmed by a GAM that showed a general increasing trend between offshore SST and distance of prey capture from the colony, which became most steep at temperatures > 19.5 °C (R^2^ = 0.46, F = 9.602, P < 0.0001) (Figure S7).

## Discussion

Southeast Australia is a hotspot for ocean warming driven by the EAC, with SST rises of 0.7–1.4 °C predicted by 2030, and 2–3 °C by 2100^28^. To better understand the ecological effects of this strengthening western boundary current, we used accelerometry in conjunction with remote-sensed environmental data to link the foraging success of a marine predator to local SST. We observed a consistent relationship between high SST and low penguin foraging success, both temporally and spatially. These findings may give important insights into resource availability in a changing system, and we discuss them below.

### Temporal variability in foraging success

Little penguins feed on a variety of low trophic level species, with “forage fish” e.g. small pelagic fish, squid and krill comprising most of their diet throughout their range^29^,^30^. Globally, forage fish are important commercial stocks, and sustain many marine predator populations^31^. These species feed on phytoplankton and zooplankton, the abundance and distribution of which are tightly linked to nutrient upwelling in boundary current systems^32^. For example, in southeast Australia where waters are generally nutrient poor, upwelling events driven by wind or the dynamic action of the EAC can enrich coastal waters^33^. These ephemeral events lead to significantly increased biomass of plankton in upwelling areas^34^, which forage fish prey on. Forage fish can be highly sensitive to changes in upwelling dynamics and to environmental conditions such as temperature, and their populations can exhibit ‘boom-bust’ dynamics^35^. Fluctuations in forage fish abundance have in turn been shown to have major effects on the productivity of predators^36^, including little penguins^37^.

Although we only studied prey capture by penguins over three breeding seasons, SST during the study period varied substantially around the long-term mean, situating our findings within a climatological context. This was due to variable dynamics of the EAC, with unusual spikes of warm water penetrating the region in the spring and summer of 2013 and 2014. We found that a simple but reliable predictor of relative foraging success in November and December was whether SST was high or low relative to the same month in the other study years, with the year with the lowest mean temperatures (2012) having the highest success and the year with the highest mean temperatures (2013) having the lowest (Fig. 2).

A broad correlation between anomalously high SST and the availability of forage fish is seen in other western boundary current systems. In the Sea of Japan, which is influenced by the warm Kuroshio Current, sardine catches are lowest when SST is high^38^. In the same region, the proportion of anchovy in the diet of rhinoceros auklets was also very low during a period of high SST^39^. As well as reducing the abundance of adult fish, high SST is related to higher mortality^40^ and lower recruitment^41^ of juvenile sardines. This suggests a potentially poor outcome for clupeoid fish species and their predators as SST rises in western boundary current regions such as southeast Australia.

Although high SST was inversely related to foraging success on a monthly scale, our modelled data showed that the functional relationship between prey capture success and SST was not linear. At the lowest SSTs, prey capture success was also low. At latitudes around Montague Island (~ −36.5 S), a major phytoplankton bloom occurs each spring that increases local chlorophyll *a* concentrations by around 150%. This bloom is driven by a seasonal increase in SST, greater availability of dissolved nitrate and silicate, and a shallowing of the mixed-layer depth: conditions that promote rapid phytoplankton growth and reproduction^42^. Reduced prey capture success occurring at low SSTs may therefore represent a period when the water is not yet warm enough to facilitate the production of high phytoplankton densities, that in turn increase local abundance of planktivorous forage fish^43^. The timing of this spring bloom is likely to be important for the breeding phenology of predators, and may explain the spring/summer breeding cycle of seabirds and seals in the study area, compared with winter breeding, which is common in western Australia.

Our models suggest an optimal offshore temperature range for prey capture success of 19–21^ ^°C, with lower success outside that range (Fig. 3). Although the SST values used in this part of the analysis provide a more general index of EAC-driven temperatures affecting the shelf rather than conditions in the precise location of penguin foraging, it is notable that this ‘thermal optimum’ mirrors established relationships between sardine (*Sardinops sagax*) catches and SST in the Gulf of California, South Africa and South Australia^32^,^44^,^45^,^46^. The area around Montague Island is the most northerly summer sardine spawning ground on the east coast of Australia, and there is a commercial sardine fishery operating in this region. Catch rates are unavailable for the study period, however landings for the ~110km region of coast incorporating the penguin foraging ground were on average 75 t per month in Sep and Nov, and 10 t per month in Dec between 1984 and 2008^47^. The importance of this area to sardines and their established distribution in relation to SST suggest that the patterns of prey capture success that we identified using accelerometry may reflect processes that govern the local availability of sardines to penguins. Future tracking studies incorporating diet analysis of predators such as seabirds, animal-borne video cameras and/or direct sampling of the prey field in relation to environmental conditions would be valuable to provide further insight into the predator-prey relationships in this system.

Our results imply that offshore SST > 21 °C is related to lower prey capture success by penguins breeding on Montague Island. In an average year, these temperatures are not experienced until January, the tail end of the little penguin’s breeding season. However, in anomalous years such as 2013, pulses of warm water arrive earlier and coincide with the peak chick-provisioning period, a time of high energetic demand^48^. Reduced food availability at crucial times in the breeding cycle is likely to have poor outcomes for breeding success and survival in range-restricted species^49^,^50^. A link between high SST and low reproductive success has been established for little penguins in other parts of their range^51^,^52^,^53^ and for some other seabird species globally^54^,^55^. Although there was no demographic study on Montague Island running concurrent to this foraging study, and the effect of the observed variation in prey availability on breeding success is therefore unknown, our findings provide some evidence that any future decrease in the fitness of meso-predators related to rising SST in the EAC system may be a function of variation in local prey availability.

### Spatial variability in foraging location

It is apparent from overlaying tracks on SST anomaly maps and from the hurdle model results that little penguin foraging tends to be focused in habitat with lower SST (Fig. 4, Table S1). Little penguins are small (~1.1 kg; 40 cm in length) and have a limited ability to assess available habitat quality relative to flying seabirds that can cover greater distances and map their environment efficiently from an aerial perspective using visual and olfactory cues^56^,^57^. It is somewhat surprising therefore, that the penguins in this study appeared to be able to reliably select the coolest habitat for foraging. Across the study period the coolest area around Montague Island tended to be inshore to the southwest, and this was the destination of almost all of the penguins tracked during this study. This persistent oceanographic feature may be influenced by the shape of the coastline at this location, which curves inwards with a prominent headland, perhaps functioning as a trap for cooler water being pushed inshore by the EAC. Heading south from the colony until encountering colder water could therefore be a risk-minimising foraging strategy for penguins, if prey abundance is predictably higher in this cooler-than-average area. Future work characterising the fine-scale oceanography in this region may shed light on the local features that enhance productivity and/or concentrate prey in certain areas.

### Spatial variability in foraging success

We found a relationship between prey capture success and SST that was not accounted for by habitat preference. Even within the cooler areas that penguins selected for foraging, and in those areas that actually contained prey, the amount of prey caught was related to SST. The coldest areas provided the highest prey capture success, and the warmest areas provided the lowest prey capture success, indicating that prey distribution may be responsive to fine-scale SST. An inverse relationship between local forage fish distribution and SST has also been observed in eastern South Africa, where sardines appear to be spatially aggregated in the coolest available habitat, pushed inshore to small patches of suitable habitat by shoreward movement of the warm Agulhas current^58^.

The penguins in this study appeared to consistently forage near the surface (<10 m). This suggests that the distribution of the prey species that they were targeting was similar throughout the study period, and that the penguins maintain a relatively consistent foraging strategy, even though prey capture success can be highly variable. In previous studies of little penguins^59^ and other diving seabirds^60^, features such as thermoclines have been identified as potential foraging cues. The relationship between the vertical distribution of forage fish and the temperature profile of the water column is generally poorly studied, including in the path of the EAC. However, as the mixed layer depth on the shelf at this time was likely to be around 20 m (CSIRO Atlas of Regional Seas (CARS); http://www.marine.csiro.au/~dunn/cars2009/) and there was no evidence of a thermocline from the tag temperature data at the depths at which penguins were catching prey, it seems that whichever prey species the penguins were primarily feeding on during this period were not consistently aggregated around such subsurface features that may be related to EAC dynamics.

Our results suggest a relationship between the distance from the colony at which penguins catch most of their prey and offshore SST, with the highest SSTs being associated with the furthest foraging distances. This was influenced by penguins travelling unusually far in December 2013 and 2014, when SST was comparatively high. Increases in foraging effort (e.g. distance travelled and dive behaviour) in response to shifts in the location of profitable feeding areas may ultimately affect population dynamics^61^. We recommend future longitudinal studies mapping the prey field using active acoustics^62^, alongside the collection of high resolution *in situ* environmental data. This will shed light on the types and densities of prey in the area at different times, and their fine-scale distribution in relation to the environment. Tracking studies of breeding seabirds could assess the ‘energy landscape’ in the region by estimating spatial gradients of energy expenditure^63^ and prey capture success from accelerometry. This could then be related back to fitness metrics such as breeding success, in order to gain more direct insight into how variability in rapidly changing ocean systems such as the EAC may affect the ability of predator populations to be sustained into the future.

## Conclusions

Marine predator populations are vulnerable to reductions in prey availability^49^,^50^ and some forage fish populations are vulnerable to rising SST^32^,^38^,^40^,^41^. We have shown that short-term variability in SST is related to prey capture success by little penguins, and that future increases in SST driven by a strengthening western boundary current may alter the abundance and distribution of forage fish. By using a prey capture signature to assess the effects of environmental variation on the relative availability of resources, we can direct future research into the way that climate change will affect species at multiple trophic levels.

## Additional Information

**How to cite this article**: Carroll, G. *et al*. High sea surface temperatures driven by a strengthening current reduce foraging success by penguins. *Sci. Rep*. **6**, 22236; doi: 10.1038/srep22236 (2016).

## Supplementary Material

Supplementary Information
