# Supplementary material for: High sea surface temperatures driven by a strengthening current reduce foraging success by penguins
Source: Sci Rep. 2016 Feb 29;6:22236. doi: 10.1038/srep22236 (PMC4770590; doi:10.1038/srep22236)
Supplement: Supplementary Information [file srep22236-s1.pdf]

Supplementary Material

Gemma Carroll, Jason D. Everett, Robert Harcourt, David Slip, Ian Jonsen

High sea surface temperatures driven by a strengthening current reduce foraging success by penguins

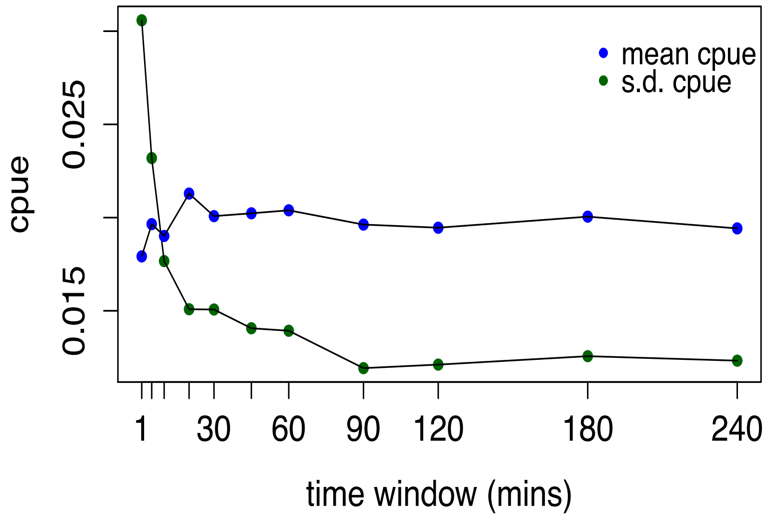

Figure S1. Mean and standard deviation of CPUE data for different time windows pooled across a subset of 8 little penguin foraging trips. 90 minutes was chosen as the most suitable window for binning data, as this showed the lowest variability between periods.

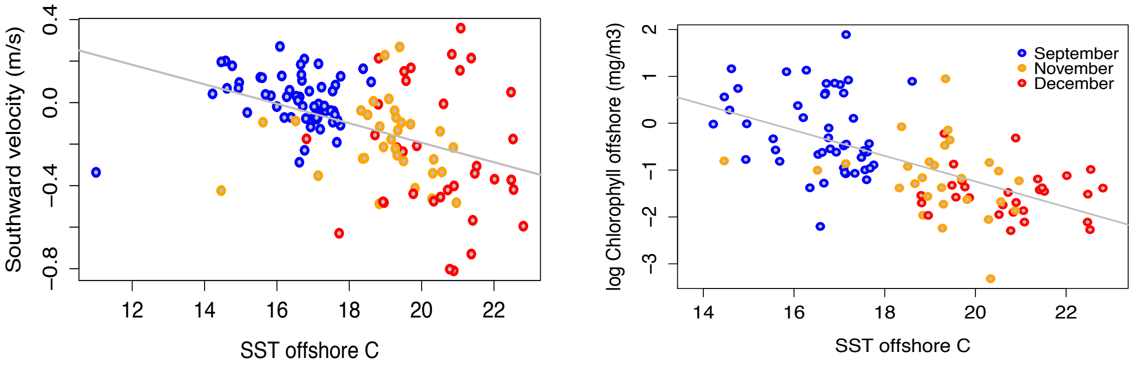

Figure S2. Relationships between southward current velocity & SST measured 5.5 km offshore from Montague Island (left) and log transformed chlorophyll concentrations offshore & SST offshore measured 5.5 km offshore from Montague Island (right)

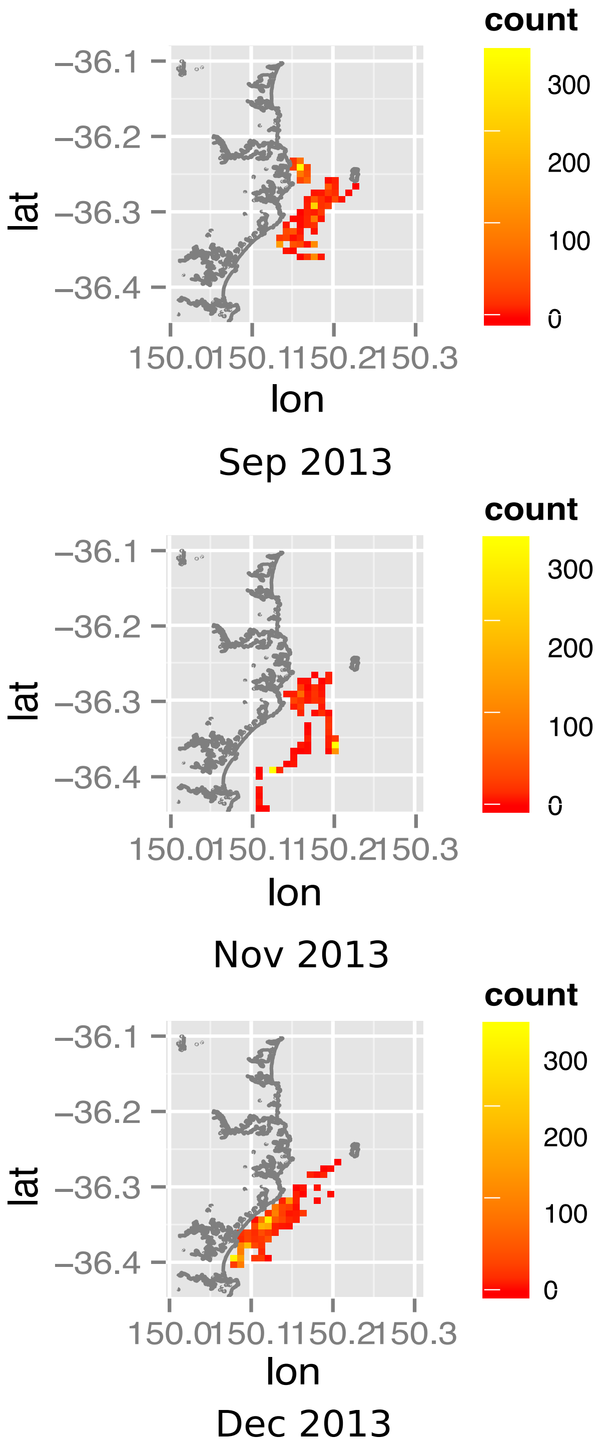

Figure S3. Map showing prey capture densities in 1km^2 grid cells during September, November and December 2013. Plot created in *ggplot2* in R version 3.2.3.

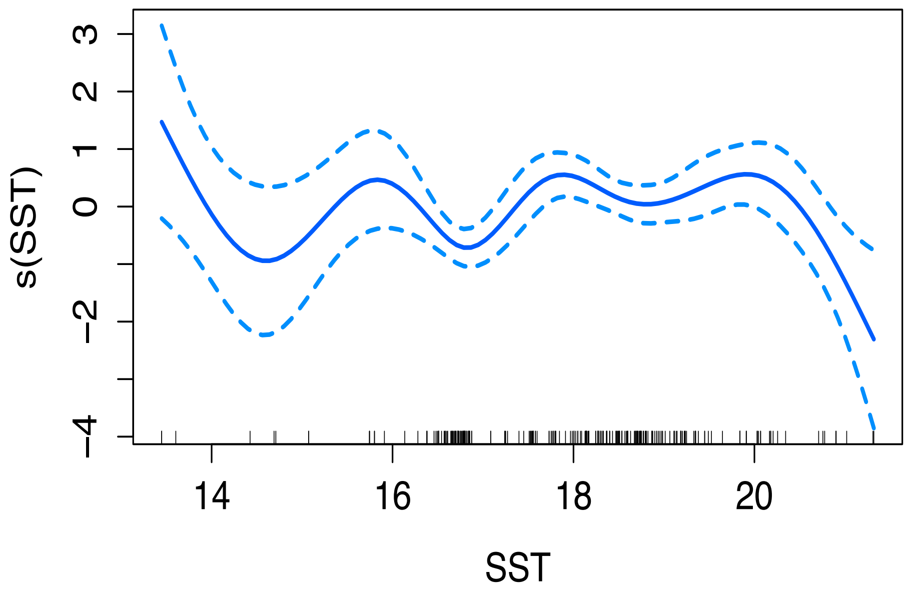

Figure S4: Generalised additive model relationship between the SST in a 1km^2 cell and the log-transformed number of prey captures in that cell. Only cells in which prey captures were recorded were included in this analysis.

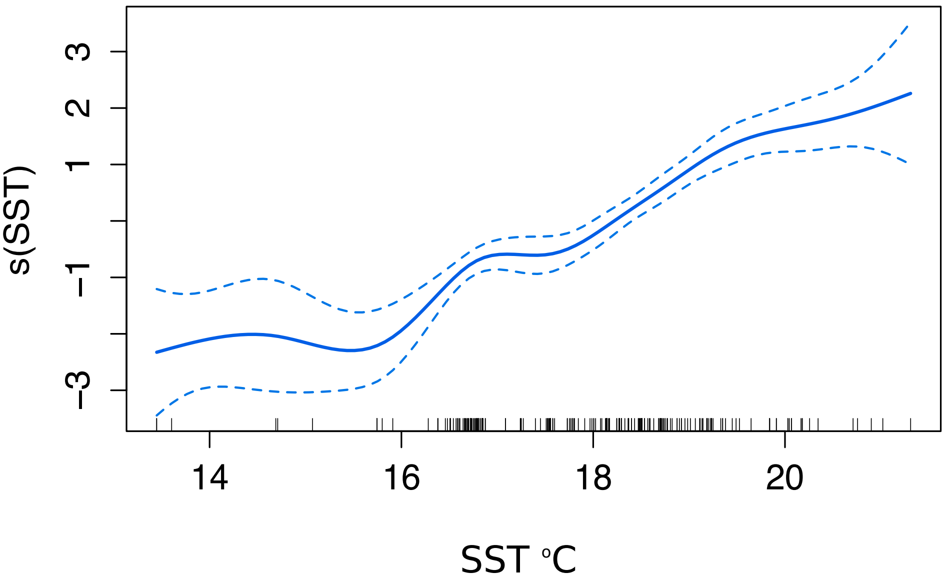

Figure S5: Generalised additive model relationship between the mean SST in a 1 km^2 grid cell and the mean temperature at which prey capture occurred in that grid cell, taken from the temperature sensor on board the accelerometer tags at the point of prey capture in the water column.

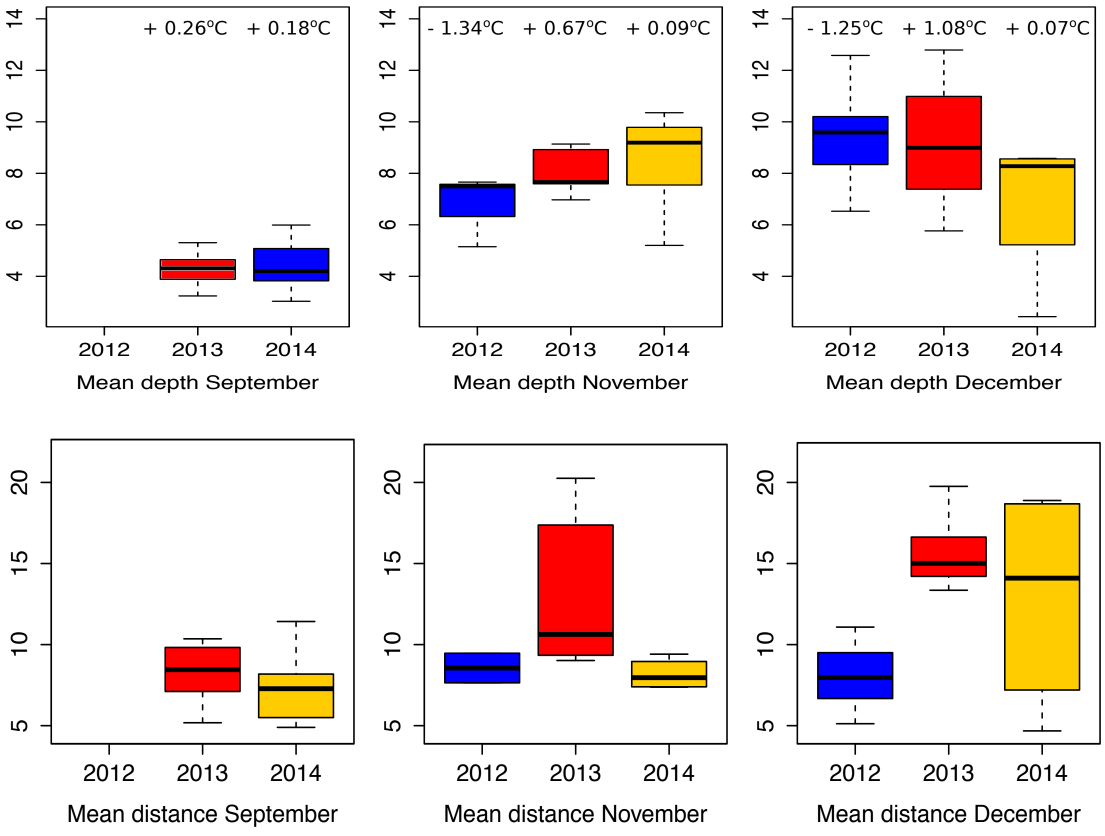

Figure S6: Boxplots showing the mean depth (m) at which prey capture occurred, and the mean distance (km) from the colony at which prey capture occurred. Box plots are coloured according to the SST relative to the mean SST of that month in the other two study years (red being the warmest of the 3 years for each month, blue being the coldest). Sample size and deviation from the mean monthly temperature (^{o}C) are noted on the top series of plots.

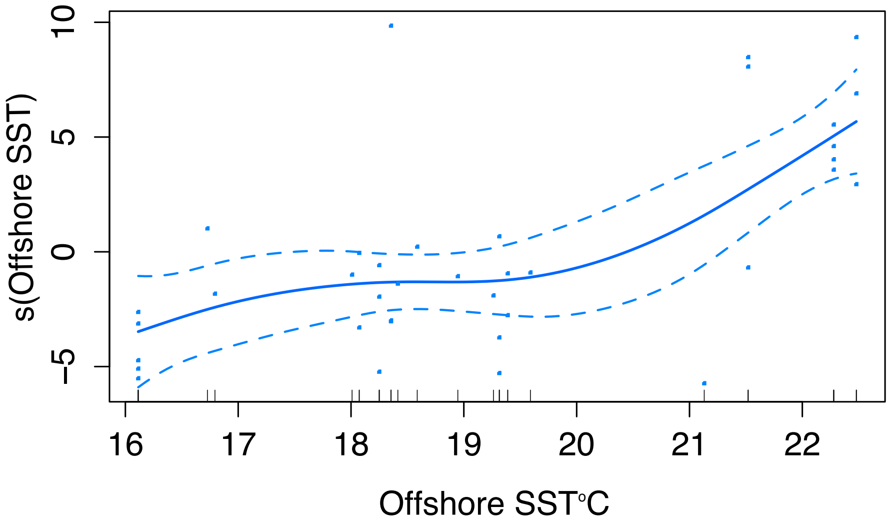

Figure S7: Generalised additive model relationship between SST recorded offshore from Montague Island and the mean distance from the colony at which penguins caught prey.

Table S1: Output from hurdle models describing the distribution of counts (number of penguin GPS locations in $1km^2$ grid cells) and the distribution of zeros (presence vs absence of penguin GPS locations in $1km^2$ grid cells) in relation to sea surface temperature (SST) and SST anomaly (both as a continuous and binary variable) in those cells.

Count model	Estimate	Std. Error	Z value	P value	AIC	Δ AIC
Intercept	2.72	0.14	19.52	< 0.0001	13398	0
SST	-0.02	0.01	-3.00	0.003		
Zero model	Estimate	Std. Error	Z value	P value		
Intercept	3.65	0.48	7.61	<0.0001		
SST	-0.28	0.03	-10.45	< 0.0001		

Count model	Estimate	Std. Error	Z value	P value	AIC	Δ AIC
Intercept	2.30	0.01	178.25	<0.0001	13440	42
SST anomaly	-0.01	0.02	-0.89	0.37		
Zero model	Estimate	Std. Error	Z value	P value		
Intercept	-1.45	0.04	-32.45	<0.0001		
SST anomaly	-0.50	0.05	-9.06	<0.0001		

Count model	Estimate	Std. Error	Z value	P value	AIC	Δ AIC
Intercept	2.21	0.02	114.43	<0.0001	13422	24
SST anomaly bin	0.17	0.02	6.62	<0.0001		
Zero model	Estimate	Std. Error	Z value	P value		
Intercept	-1.72	0.06	-27.18	<0.0001		
SST anomaly bin	0.66	0.09	7.56	<0.0001		
